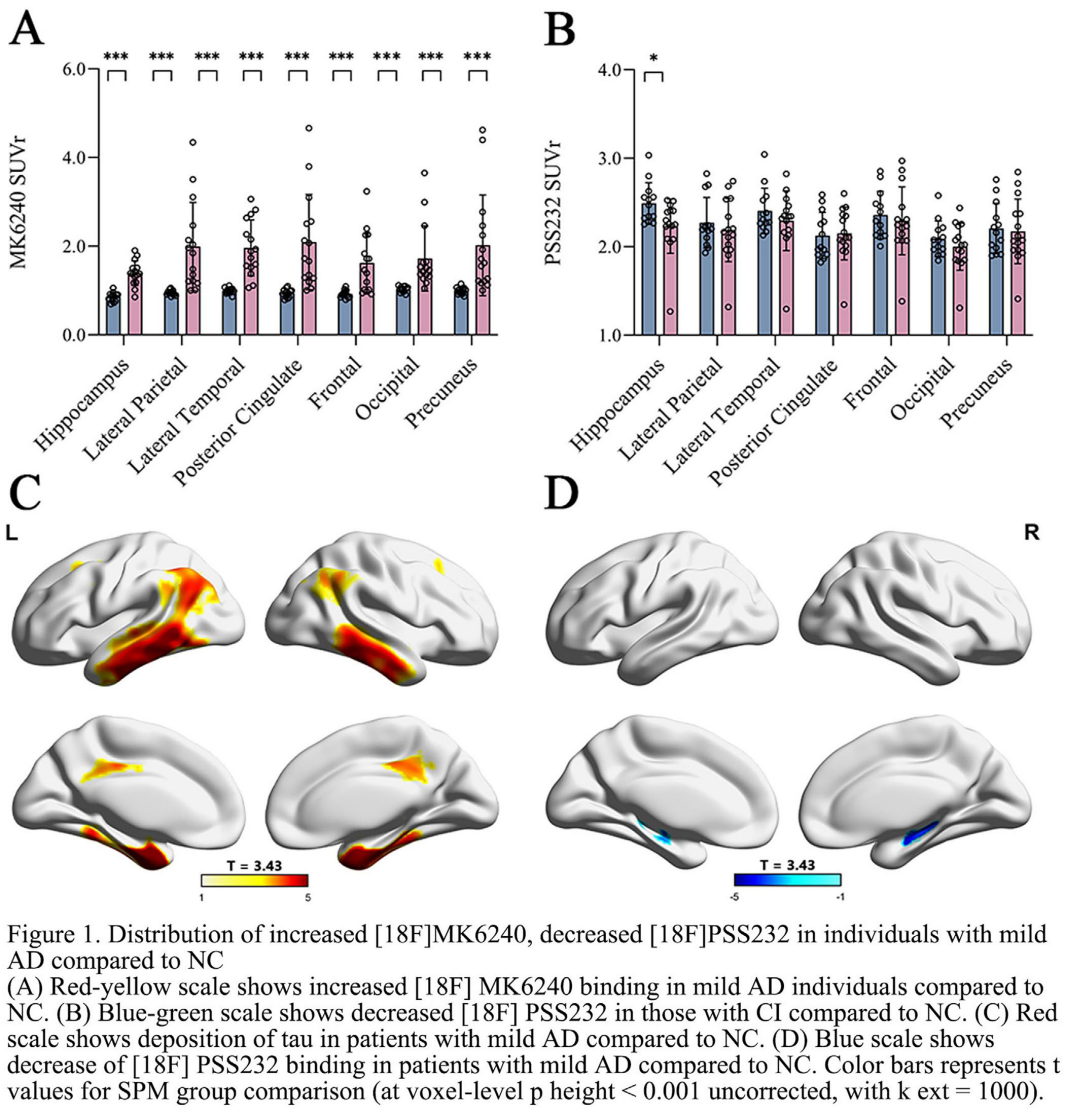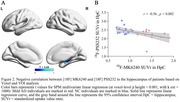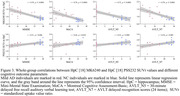# Metabolic glutamate receptor 5 (mGluR5) is associated with tau pathology in mild Alzheimer's disease

**DOI:** 10.1002/alz.093799

**Published:** 2025-01-09

**Authors:** Yan Wang, Fang Xie, Yihui Guan

**Affiliations:** ^1^ Huashan Hospital, Fudan University, Shanghai, Shanghai China

## Abstract

**Background:**

Alzheimer's disease (AD) is characterized by the deposition of amyloid plaques and tau neurofibrillary tangles in the brain with continuous cognitive impairment. Although the mechanism underlying AD pathogenesis remains unclear, more evidence suggests that synaptic dysfunction and loss may be an early event in disease progression. Metabotropic glutamate receptor 5 (mGluR5), a kind of G protein–coupled receptor, is involved in AD pathology through modulating synaptic transmission and plasticity and thus exhibits therapeutic effects. However, research on the associations of mGluR5 with the neuropathological hallmarks of AD needs to be completed.

**Method:**

In this single‐center, cross‐sectional study, fifteen patients with mild AD and thirteen normal controls (CN) underwent a PET/MRI scan with [18F] PSS232 (mGluR5 availability) and trouble PET/CT scan with [18F] florbetapir (ß‐amyloid) and [18F] MK6240 (tau deposition). The associations of [18F] PSS232 and [18F] MK6240 binding were investigated. We also investigated the correlations of tau deposition and mGluR5 availability with cognitive performance. Voxel‐wise and volume‐of‐interest (VOI) analyses were performed on PET data.

**Result:**

Compared to CN, individuals with mild AD showed smaller clusters of [18F] PSS232 binding in the hippocampus and increased tau deposition in the hippocampus, lateral temporal, posterior cingulate, frontal, occipital, and parietal (Figure 1). Based on Voxel‐wise and VOI analyses, tau deposition in the hippocampus was negatively associated with [18F] PSS232 binding in the hippocampus (r = 0.56, p = 0.002, Figure 2). Lower neuropsychological test scores were associated with decreased [18F] PSS232 and increased [18F] MK6240 binding in the hippocampus (Figure 3).

**Conclusion:**

Patients with mild AD have significant reductions in mGluR5 availability and have high tau deposition in the hippocampus, which is considered the critical region involved in early cognitive impairment. That means mGluR5 and tau were interrelated in the hippocampus and may apply in tau spreading. The correlation of mGluR5 availability and tau deposition in severe AD patients and more longitudinal data are needed to illustrate the role of mGluR5 in AD pathology.